# Oncogenic AKTivation of translation as a therapeutic target

**DOI:** 10.1038/bjc.2011.241

**Published:** 2011-07-19

**Authors:** A C Hsieh, M L Truitt, D Ruggero

**Affiliations:** 1 Department of Urology, School of Medicine, Helen Diller Family Comprehensive Cancer Center, University of California, San Francisco, Helen Diller Family Cancer Research Building, Room 386, 1450 3rd Street, San Francisco, CA 94158-3110, USA; 2 Division of Hematology/Oncology, University of California, San Francisco, Helen Diller Family Cancer Research Building, Room 386, 1450 3rd Street, San Francisco, CA 94158-3110, USA

**Keywords:** AKT, eIF4E, translational control, ribosome, PI3K, mTOR

## Abstract

The AKT signalling pathway is a major regulator of protein synthesis that impinges on multiple cellular processes frequently altered in cancer, such as proliferation, cell growth, survival, and angiogenesis. AKT controls protein synthesis by regulating the multistep process of mRNA translation at every stage from ribosome biogenesis to translation initiation and elongation. Recent studies have highlighted the ability of oncogenic AKT to drive cellular transformation by altering gene expression at the translational level. Oncogenic AKT signalling leads to both global changes in protein synthesis as well as specific changes in the translation of select mRNAs. New and developing technologies are significantly advancing our ability to identify and functionally group these translationally controlled mRNAs into gene networks based on their modes of regulation. How oncogenic AKT activates ribosome biogenesis, translation initiation, and translational elongation to regulate these translational networks is an ongoing area of research. Currently, the majority of therapeutics targeting translational control are focused on blocking translation initiation through inhibition of eIF4E hyperactivity. However, it will be important to determine whether combined inhibition of ribosome biogenesis, translation initiation, and translation elongation can demonstrate improved therapeutic efficacy in tumours driven by oncogenic AKT.

## Translational Control by AKT

**Figure 1:**
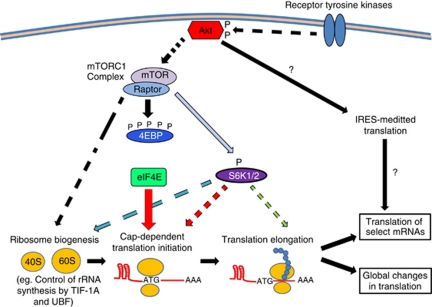
AKT signalling coordinately regulates translation. AKT is activated downstream of various cellular and oncogenic stimuli, such as receptor tyrosine kinase (RTK) signalling, to promote protein synthesis. AKT may accomplish this through coordinated regulation of ribosome biogenesis, translation initiation, and translation elongation. AKT-driven protein synthesis requires a full repertoire of mature ribosomes, and AKT has been shown to promote ribosome biogenesis through both enhanced rRNA synthesis and enhanced ribosomal protein production. In addition, AKT promotes protein synthesis through the activation of translation initiation factors that drive cap-dependent translation. This is one of the most rapid mechanisms by which AKT can activate protein synthesis, and it occurs largely through mTORC1-dependent phosphorylation of the 4EBPs. Furthermore, AKT has been shown to affect the efficiency of translation through the control of translation elongation factors. Translation can also be regulated through additional mechanisms, such as IRES-mediated translation, and it remains to be seen what effect AKT signalling may have on these processes. Together, AKT regulates the multiple stages of translation to drive both global changes in protein synthesis as well as selective changes in the translation of specific mRNAs.

Protein synthesis is one of the most costly and tightly regulated energetic investments downstream of AKT signalling. AKT regulates protein synthesis through the phosphorylation of multiple downstream targets that function together to control all stages of mRNA translation from ribosome biogenesis to translation initiation and elongation (Figure 1). Ribosome biogenesis, translation initiation, and translation elongation are all frequently deregulated in cancer, and it is likely that oncogenic AKT drives tumour development and progression in part through its ability to coordinately activate these various steps of the translational process.

### AKT activates translation initiation

One of the most rapid ways that AKT signalling enhances protein synthesis is through the activation of translation initiation. Translation initiation is the process by which ribosomes are recruited to the 5′ untranslated region (5′ UTR) of mature mRNAs in the first step of protein synthesis. In this process, 40S ribosomal subunits are recruited to the 7-methyl guanosine cap (5′ cap) of mRNAs by the eIF4F translation initiation complex through interactions with eukaryotic initiation factor 3 (eIF3; Emanuilov *et al*, 1978). eIF4F is a trimeric complex that resides at the cap. It is composed of the 5′ cap mRNA-binding protein eIF4E, the RNA helicase eIF4A, and the scaffolding molecule eIF4G (Haghighat and Sonenberg, 1997; Rogers *et al*, 1999). The majority of mRNA translation begins through eIF4F association with the cap and is known as cap-dependent translation. Translation initiation is considered to be the rate-limiting step of cap-dependent translation. eIF4E is considered as the key factor in controlling this step (Duncan *et al*, 1987). This thought is based largely on the fact that eIF4E activity is highly regulated at both the mRNA and protein level. eIF4E is upregulated at the mRNA level by a number of transcription factors including the oncogene MYC (Jones *et al*, 1996). At the protein level, eIF4E activity is controlled through an activating phosphorylation at serine 209, as well as through inhibitory interactions with the eIF4E-binding proteins (4EBPs; Gingras *et al*, 1998; Topisirovic *et al*, 2004). This tight regulation of eIF4E activity provides a rapid mechanism for cells to modulate translation initiation in response to numerous stimuli, including growth factor and oncogenic signalling.

**Figure 2:**
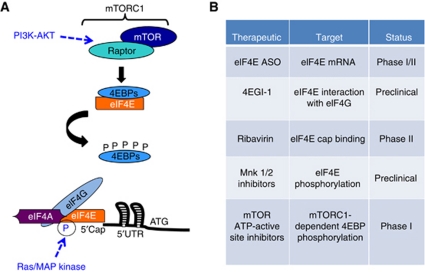
Targeting eIF4E hyperactivation in cancer. (**A**) Oncogenic AKT signalling promotes translation initiation predominantly through mTORC1-dependent hyperactivation of eIF4E. In the absence of signalling, hypophosphorylated 4EBP binds to and inhibits eIF4E, blocking its ability to interact with eIF4G. AKT signalling activates mTORC1, initiating a series of phosphorylations that release 4EBP from eIF4E. This allows for eIF4G binding to eIF4E and the subsequent recruitment of the 40S ribosomal subunit. In addition, it has been shown that Ras/MAP kinase signalling can promote eIF4E hyperactivation through downstream phosphorylation of eIF4E at Serine 209. (**B**) Current clinical status and proposed mechanistic targets of therapeutics designed to inhibit eIF4E hyperactivation in cancer.

AKT controls translation initiation largely through activation of the kinase mammalian target of rapamycin complex 1 (mTORC1). mTORC1 phosphorylates ribosomal protein (RP) S6 kinase 1/2 (S6K1/2) and the 4EBPs (Brown *et al*, 1995; von Manteuffel *et al*, 1997; Gingras *et al*, 1998). The 4EBPs are a family of small proteins (4EBP1–3) that compete with eIF4G for binding to the dorsal surface of eIF4E. In a hypophosphorylated state, 4EBPs prevent the formation of the eIF4F complex on the 5′ UTR of mRNAs by binding to eIF4E and preventing eIF4G recruitment (Figure 2). However, on growth factor stimulation, 4EBPs are phosphorylated at multiple serine/threonine residues in a series initiated by mTORC1 (Gingras *et al*, 1999, 2001). This leads to a conformational change that releases 4EBPs from eIF4E and allows eIF4G to bind eIF4E and ultimately recruit the 40S ribosomal subunit to the 5′ end of mRNAs. As a result, eIF4E regulates global protein synthesis by controlling the rate that ribosomes are able to dock onto the 5′ cap of mRNAs.

In addition to global increases in protein synthesis, eIF4E hyperactivation is able to enhance the translation of select mRNAs (Mamane *et al*, 2007). The 5′ UTR of these mRNAs are believed to be the regulatory factors that impart this selectivity. 5′ UTRs can vary in length and GC nucleotide content, resulting in a range of secondary mRNA structures. These structures function as physical barriers that limit the ability of the 40S ribosome to reach the translation start site (Manzella and Blackshear, 1990). As such, mRNAs with complex 5′ UTRs have low basal rates of translation and are exquisitely sensitive to eIF4E hyperactivation due to the ability of eIF4E to recruit the eIF4A helicase. eIF4A recruitment allows for enhanced unwinding of secondary structures in the 5′ UTR, resulting in improved translation initiation efficiency. Genes that are sensitive to eIF4E-mediated translation cover a range of cellular functions, including cell cycle control (cyclin D1), angiogenesis (VEGF), metabolism (ODC), and apoptosis (survivin and Mcl-1) among others (Rousseau *et al*, 1996; Graff *et al*, 2007; Mamane *et al*, 2007; Mills *et al*, 2008).

Despite the identification of mRNA targets that rely on the eIF4F complex for efficient translation (see above), several unbiased screens have also identified transcripts that lack complex 5′ UTRs, but are sensitive to eIF4E hyperactivation (Larsson *et al*, 2006; Mamane *et al*, 2007). One such class of genes is the 5′ TOP genes. 5′ TOP genes (terminal oligopyrimidine or tract of oligopyrimidine genes) are characterised by oligopyrimidine repeats in the 5′ UTR and predominantly encode for RPs, translation initiation factors, and translation elongation factors (Levy *et al*, 1991; Avni *et al*, 1994, 1997). While it is still unknown how hyperactivated eIF4E specifically regulates the translation of 5′ TOP genes, the fact that these genes do not possess complex 5′ UTRs suggests that there are other mechanisms of translational regulation downstream of eIF4E that have not been described. How might these additional regulatory mechanisms occur? One possible mechanism by which eIF4E could regulate translation is through direct interaction with inhibitory mRNA secondary structures outside of the 5′ UTR. eIF4E hyperactivation could promote unwinding of these structures through the recruitment of the eIF4A helicase, allowing for mRNA translation. In support of this, it was recently shown that eIF4E binds to a specific secondary structure outside of the 5′ UTR of Histone H4 mRNA to promote the translation of this mRNA in an eIF4A-dependent manner (Martin *et al*, 2011).

To elucidate how unique mRNA secondary structures interact with the translation initiation complex and the scanning ribosome, it will require the development of techniques to determine simultaneously the position of ribosomes on mRNAs and the precise secondary structures at that particular location. There are now rising technologies that may allow for this. In particular, the ability to deep-sequence ribosome-protected mRNAs has enabled us to determine the precise location of actively translating ribosomes (Ingolia *et al*, 2009). Furthermore, through deep sequencing, it is also now possible to determine the secondary structures of mRNAs by using parallel analysis of secondary structures (Kertesz *et al*, 2010). The combination of these two technologies may provide a very accurate portrait of how mRNA secondary structures control cap-dependent translation and allow for the identification of translational networks of genes with common regulatory elements within their mRNAs.

### AKT promotes translation elongation

Although significant attention has been focused on the ability of AKT to regulate translation initiation, evidence suggests that other steps of translation, such as translation elongation, are also regulated by AKT signalling. Translation elongation is the process by which amino acid-charged tRNAs dock onto the ribosome/mRNA complex and incorporate amino acids into the growing nascent polypeptide chain. Multiple elongation factors are necessary to carry out this process efficiently. The eukaryotic translation elongation factor 2 (eEF2) mediates the translocation step of elongation, where tRNAs move between the P and A site on the ribosome as the ribosome migrates by one codon along the mRNA. AKT can promote this elongation step through S6K1/2-dependent inhibition of eEF2 kinase, a negative regulator of eEF2 (Wang *et al*, 2001). Thus, AKT activation not only affects translation initiation but also the efficiency of actively translating ribosomes. In addition, there is evidence that AKT activation may more broadly impact translation elongation through the preferential translation of 5′ TOP genes (see above), many of which encode for translation elongation factors (Avni *et al*, 1997; Mamane *et al*, 2007). While many mechanistic gaps still exist, it will be important to understand the degree to which AKT-activated translation elongation can enhance protein synthesis. Furthermore, it needs to be established if this ability of AKT to modulate translation elongation can contribute to AKT-driven tumourigenesis. It is interesting to speculate that oncogenic AKT may promote translation elongation not to cause increases in global protein synthesis but to instead preferentially promote the translation of select mRNAs.

### AKT controls ribosome biogenesis

Protein synthesis depends on the generation of properly assembled, mature ribosomes. The biogenesis of mature ribosomes involves the synthesis and processing of rRNA, the synthesis of RPs, and the proper assembly of all these components within the nucleolus. AKT has been shown to modulate various aspects of these processes predominantly through mTOR activation. For example, mTOR can enhance the transcription of rDNA through activation of transcription initiation factor 1A (TIF-1A), a RNA polymerase I (Pol I) transcription factor. Through an indirect mechanism, mTOR simultaneously promotes an activating phosphorylation and blocks an inhibitory phosphorylation of TIF-1A to enhance rRNA synthesis (Mayer *et al*, 2004). In addition, mTOR can promote rRNA synthesis through activation of another Pol I transcription factor, upstream binding factor (UBF). Although the precise mechanism behind mTOR-dependent UBF activation has not been identified, S6K1 is thought to be required for UBF activation (Hannan *et al*, 2003). While AKT has been shown to enhance rRNA synthesis through multiple mechanisms, the ability of AKT to regulate other ribosomal constituents is less well defined. AKT activation may promote RP synthesis through enhanced translation of 5′ TOP genes (see above), which include many RPs. In addition, it has been demonstrated in yeast that RP synthesis is positively regulated by mTOR. In this setting, mTOR promotes the transcription of RP genes by indirectly activating transcription factors such as FHL1 (Martin *et al*, 2004). Despite evidence that AKT signalling can regulate ribosomal biogenesis through modulation of both rRNA synthesis and RP synthesis, the connection between ribosomal biogenesis and protein synthesis remains poorly defined. While studies have shown that normal ribosomal biogenesis is in fact required for protein synthesis, it is not clear if enhanced ribosomal biogenesis is able to drive increased protein synthesis downstream of oncogenic AKT.

### AKT and IRES-mediated translation

Another mechanism of initiating translation, which may be targeted by AKT, is internal ribosome entry site (IRES)-mediated translation. IRES elements are mRNA secondary structures predominantly located within the 5′ UTR (and to a lesser extent in the coding sequence and intronic regions of mRNA) that can associate with IRES *trans*-acting factors (ITAFs) to initiate translation in a 5′ cap and eIF4E-independent manner. Only a subset of mRNAs contains IRES sequences. Thus, IRES-mediated translation is thought to be a fine-tuning mechanism that controls the translation of key mRNAs under specific physiological conditions such as the G0/quiescent and G2/M phases of the cell cycle, where it modulates proliferation, as well as under specific stress conditions such as hypoxia, where it promotes cell survival and angiogenesis (Pyronnet *et al*, 2000; Miskimins *et al*, 2001; Kullmann *et al*, 2002; Braunstein *et al*, 2007). IRES-mediated translation has been shown to have a role in cancer. One example for this is in the setting of hypoxia in invasive breast cancer, where it has been demonstrated in a mouse model that eIF4E-mediated translation is downregulated through increased expression of 4EBP1 under hypoxic conditions. Despite a resulting decrease in overall protein synthesis levels, specific IRES containing mRNAs such as VEGF, HIF-1*α*, and Bcl-2 are translated at higher rates, thereby increasing the protein levels of these protumourigenic targets (Braunstein *et al*, 2007). In this manner, IRES-mediated translation enhances survival under specific cellular conditions.

Recently, there has been intriguing evidence that AKT may regulate IRES-mediated translation at the level of ITAFs. In particular, AKT was shown to directly phosphorylate the ITAF hnHRP1A at serine 199 and inhibit IRES-mediated translation (Jo *et al*, 2008). In this way, AKT may actively limit IRES-mediated translation through phosphorylation of ITAFs while it simultaneously promotes cap-dependent translation (see above). Further studies will be needed to delineate the role that inhibition of IRES-mediated translation has in AKT-driven tumour development and progression.

AKT signalling functions as a critical node for mRNA translation, coordinating everything from ribosome biogenesis to translation initiation and elongation. AKT collectively activates these stages of translational control to drive increased cellular protein synthesis. Despite strong evidence that AKT signalling can regulate ribosome biogenesis, translation initiation, and translation elongation, we still do not understand the relative role that these events have in AKT-induced protein synthesis. Future studies will be needed to determine the extent to which activation of these translational steps, either alone or in combination, is sufficient to drive protein synthesis. In addition, it will be important to understand the requirement for activation of each of these translational stages in tumours driven by oncogenic AKT signalling. Despite the fact that AKT functions as a master regulator of translational control through mTOR activation, AKT also has other well-characterised targets, including but not limited to FOXO, GSK3, and MDM2, which control diverse cellular processes, such as cell survival, proliferation, and angiogenesis, without directly impinging on mRNA translation (Manning and Cantley, 2007). For example, AKT-mediated phosphorylation and activation of MDM2 leads to p53 ubiquitination, degradation, and significantly impairs the cellular DNA damage response (Zhou *et al*, 2001). Thus, oncogenic AKT exhibits its transforming potential through multiple mechanisms. Intriguingly, several downstream effectors of mTOR-independent AKT targets have been shown to be translationally regulated. For example, AKT promotes cell survival in part through inhibition of GSK3, preventing the phosphorylation and subsequent degradation of the prosurvival Bcl-2 family member Mcl-1 (Maurer *et al*, 2006), and Mcl-1 has also been shown to be translationally upregulated downstream of oncogenic AKT signalling (Hsieh *et al*, 2010). Therefore, it remains an open and outstanding question to what extent AKT-mediated translational control cooperates with key non-mTOR-dependent AKT substrates to regulate critical cellular events to promote tumourigenesis and cancer progression.

## Translation initiation is critically required for oncogenic AKT activity

The AKT signalling pathway is heavily mutated in a variety of human malignancies. In fact, mutations of AKT pathway components and upstream regulators cover nearly the entire spectrum of human cancers, suggesting a broad requirement for AKT activation in tumourigenesis (Table 1). Although genetic alterations of AKT are relatively rare in human cancers, multiple mouse studies have demonstrated that the expression of constitutively active AKT isoforms is sufficient to drive tumourigenesis (Mende *et al*, 2001; Majumder *et al*, 2003; Tan *et al*, 2008). Furthermore, AKT hyperactivity has been shown to be critically required for tumourigenesis caused by more frequently occurring genetic lesions upstream of AKT signalling, such as PTEN loss (Chen *et al*, 2006). Despite a wealth of knowledge on genetic mutations leading to the oncogenic activation of AKT and a growing appreciation for the ability of AKT to coordinately regulate mRNA translation, the extent to which deregulated AKT translational control functions as an oncogenic driver remains largely undefined. Recent studies have, however, highlighted a critical requirement for enhanced translation initiation downstream of oncogenic AKT signalling. Strikingly, oncogenic AKT seems to enhance translation initiation largely through hyperactivation of the eIF4E translation initiation factor, which is a bona-fide oncogene.

The oncogenic potential of eIF4E has been well described both *in vitro* and *in vivo*. Overexpression of eIF4E is sufficient to induce transformation of fibroblasts and primary epithelial cells in culture, and eIF4E overexpression in mice leads to increased cancer susceptibility in a range of tissues (Lazaris-Karatzas *et al*, 1990; Avdulov *et al*, 2004; Ruggero *et al*, 2004). While these findings, along with evidence of eIF4E overexpression in human cancers (Flowers *et al*, 2009; Graff *et al*, 2009; Wang *et al*, 2009), support the notion that eIF4E is oncogenic, a direct connection between eIF4E and translational deregulation downstream of oncogenic AKT signalling has only recently been described. Some of the first evidence for such a connection came from a study showing that pharmacological inhibition of oncogenic RAS and AKT in glioblastoma cells caused a rapid and profound change in mRNA translation that far outweighed transcriptional changes and was associated with loss of mTORC1-dependent phosphorylation of 4EBPs (Rajasekhar *et al*, 2003). This study identified translational regulation of several mRNA targets important for cancer development, and suggested that altered translational control downstream of eIF4E hyperactivation may be required for AKT-driven cellular transformation.

Our group demonstrated *in vivo* that hyperactivation of eIF4E is necessary for AKT-mediated tumourigenesis. Using a T-cell lymphoma model driven by overexpression of constitutively active AKT, we showed that enhanced protein synthesis through eIF4E hyperactivation was required for AKT-mediated tumourigenesis. We found that AKT overexpressing pretumour progenitor T cells possessed a distinct survival advantage, which was abrogated when eIF4E hyperactivity was restored to wild-type levels. Using a candidate gene approach, we found that this survival advantage was due in part to translational upregulation of the antiapoptotic Mcl-1. Importantly, we were also able to pharmacologically inhibit eIF4E hyperactivity downstream of oncogenic AKT, which resulted in significant inhibition of tumour growth (see below; Hsieh *et al*, 2010). As such, we identified the 4EBP/eIF4E axis as a druggable target that regulates translation downstream of oncogenic AKT.

The requirement for eIF4E hyperactivity in AKT-driven tumours has been further substantiated by recent studies. For example, it was found that the efficacy of an AKT inhibitor in human cancer cell lines correlated with its ability to inhibit phosphorylation of 4EBPs and block cap-dependent translation. This study showed that in cell lines where AKT inhibition failed to block phosphorylation of 4EBPs, the MAPK signalling pathway was frequently activated. The authors further demonstrated that combined pharmacological inhibition of AKT and MAPK signalling was able to inhibit phosphorylation of 4EBPs and prevent the *in vivo* growth of cell lines resistant to AKT inhibition alone. Importantly, the authors were able to attribute this combinatorial drug effect directly to the inhibition of eIF4E hyperactivity, as the overexpression of a non-phosphorylatable form of 4EBP1 was sufficient to block the growth of these cells in xenografts (She *et al*, 2010).

In addition to 4EBP-dependent control, eIF4E activity is positively regulated through phosphorylation at serine 209 by the MAP kinase targets MNK1/2. Whole body expression of a knock-in mutant of eIF4E, which can no longer be phosphorylated at this residue, was found to decrease the incidence and grade of prostatic intraepithelial neoplasia in a mouse prostate cancer model driven by PTEN loss (Furic *et al*, 2010). While this study supports a role for eIF4E hyperactivation downstream of oncogenic AKT signalling, it raises several questions: Do all tissues rely on phosphorylation of serine 209 for hyperactivation of eIF4E downstream of oncogenic AKT signalling? More broadly, what is the tissue-specific dependence of eIF4E hyperactivation, which could be achieved by different mechanisms, downstream of oncogenic AKT? Indeed, there is convincing genetic evidence that oncogenic eIF4E alone is sufficient to drive tumourigenesis in specific tissues. Transgenic mice that ubiquitously overexpress eIF4E show that distinct tissues, including the lungs, liver, and the lymphoid compartment, are more prone to oncogenic transformation (Ruggero *et al*, 2004). As such, we can speculate that there may be tissue-specific requirements for the eIF4E oncogenic activity downstream of AKT hyperactivation in tumour development. Although many important questions remain to be addressed, the above studies show that eIF4E hyperactivation is not only critically required for AKT-driven tumours but it might also serve as a node on which multiple oncogenic signalling pathways converge, thus representing an attractive therapeutic target.

## Targeting eIF4E hyperactivation

### Antisense targeting of eIF4E – eIF4E ASO

eIF4E is a bona-fide oncogene frequently hyperactivated downstream of oncogenic AKT signalling, and thus represents an attractive target for rational drug design. There are currently several approaches being pursued to therapeutically inhibit eIF4E, but perhaps the most direct of these approaches is the use of specific antisense oligonucleotides (ASOs) that bind to eIF4E mRNA and mediate its destruction by RNase H. Nanomolar concentrations of eIF4E ASOs have been shown to decrease eIF4E protein levels in several human cancer cell lines *in vitro*, reducing protein levels of known eIF4E targets and inducing apoptosis. In tumour xenograft models, eIF4E ASOs inhibited tumour growth without any detectable changes in body weight or liver function. Strikingly, control mice treated with eIF4E ASOs for 3 weeks showed no signs of toxicity, despite reductions in eIF4E protein levels by up to 80% in the liver, implying a critical difference in the requirement of eIF4E for normal physiological function (Graff *et al*, 2007). These studies suggest that tumours may be sensitive to eIF4E inhibition while normal tissues are not, but for what duration and to what extent eIF4E can be inhibited system-wide without detriment remains an open question.

### eIF4E–eIF4G interaction inhibitor – 4EGI-1

Additional attempts to target eIF4E have focused on blocking its ability to interact with eIF4G. The interaction between eIF4E and eIF4G is dependent on an eIF4G Y(X)_4_LΦ motif, where X is variable and Φ is hydrophobic (Altmann *et al*, 1997). High-throughput screens for inhibitors that could prevent eIF4E binding to the Y(X)_4_LΦ motif identified 4EGI-1 as a candidate compound. 4EGI-1 was able to inhibit eIF4F complex formation at micromolar concentrations. Surprisingly, 4EGI-1 did not block the ability of eIF4E to bind to 4EBP1, which, similar to eIF4G, contains a Y(X)_4_LΦ motif. 4EGI-1 was shown to be cytostatic and cytotoxic in multiple cell lines and preferentially blocked the growth of transformed cells over untransformed cells (Moerke *et al*, 2007). Recently, it has been reported that 4EGI-1 functions through an eIF4G/eIF4E-independent mechanism to promote apoptosis in human lung cancer cells (Fan *et al*, 2010). Additionally, 4EGI-1 has been shown to suppress translation in primary human cells at concentrations below those required for eIF4E inhibition (McMahon *et al*, 2011). Collectively, these studies suggest that 4EGI-1 may have antitumour efficacy through more general inhibition of oncogenic pathways and that the full spectrum of protein–protein interactions and pathways that 4EGI-1 blocks still needs to be determined. Despite these concerns, specifically targeting the eIF4E/eIF4G protein–protein interaction is an attractive therapeutic approach, and subsequent generations of such inhibitors may provide a novel and important way of targeting eIF4E in human cancers.

### Targeting the eIF4E-5′ cap interaction – Ribavirin

eIF4E function can also be directly inhibited by blocking its ability to interact with the 5′ cap of mRNAs. Ribavirin, a guanosine ribonucleoside currently used as an anti-viral therapy, has recently been shown to compete with endogenous mRNAs for binding to eIF4E, leading to decreased eIF4F complex formation *in vitro*. In line with this, ribavirin blocked eIF4E-mediated oncogenic transformation *in vitro* and demonstrated *in vivo* efficacy in preclinical models of acute myelogenous leukaemia (AML) and squamous cell carcinoma (Kentsis *et al*, 2004). In a phase I dose-escalation trial with ribavirin, 7 out of 11 AML patients were reported to have at least partial responses or stable disease (Assouline *et al*, 2009). Although ribavirin may ultimately prove to have clinical efficacy in human cancers, the specific function of ribavirin as a cap-mimetic has been called into question by two independent groups (Westman *et al*, 2005; Yan *et al*, 2005). Therefore, it is not clear if the inhibition of cap-dependent translation underlies ribavirin's therapeutic efficacy.

### Inhibition of eIF4E phosphorylation – MNK kinase inhibitors

The MNK kinases are activated downstream of MAP kinase signalling and directly phosphorylate eIF4E at serine 209 (Scheper *et al*, 2001). Mutation of eIF4E at this residue blocks its transforming potential *in vitro* and can inhibit PTEN-driven tumourigenesis *in vivo* (Furic *et al*, 2010). Furthermore, mice doubly deficient for MNK1 and MNK2 are resistant to lymphomagenesis driven by PTEN loss, validating the MNKs as potential therapeutic targets upstream of eIF4E (Ueda *et al*, 2010). Recently, a high-throughput screen identified the antifungal cercosporamide as a potent inhibitor of MNK1 and MNK2 with limited activity towards other kinases. Cercosporamide was able to block eIF4E phoshorylation *in vivo* and inhibit the growth of human xenografts as well as the metastasis of mouse melanoma cells (Konicek *et al*, 2011). Although these results are promising and suggest that targeted inhibition of eIF4E phosphorylation may be a valid therapeutic approach, it remains unclear to what extent the efficacy of MNK kinase inhibitors can be attributed to their ability to block other downstream phosphorylation targets critical for tumour growth and maintenance. Regardless, the observation that both MNK kinase activity and eIF4E phosphorylation are dispensable for normal growth and development, but are required for tumourigenesis, makes the MNK kinases attractive therapeutic targets.

### mTOR ATP active-site inhibitors

Perhaps one of the most promising approaches to therapeutically block eIF4E hyperactivity is the targeted inhibition of the mTOR kinase. First-generation allosteric mTOR inhibitors such as rapamycin, RAD001, and CCI-779 inconsistently inhibit phosphorylation of 4EBP1 downstream of mTORC1, despite potently inhibiting S6K phosphorylation (Choo *et al*, 2008; Hsieh *et al*, 2010). This suggests that phosphorylation of S6K or its downstream target rpS6 may not serve as an accurate readout for inhibition of all mTORC1 kinase activity. Indeed, the poor clinical performance of rapamycin and its associated analogues in human cancer is most likely due to their inability to block mTORC1-dependent phosphorylation of 4EBPs and thus fully inhibit eIF4E activation (see above). In order to overcome the incomplete inhibition seen with allosteric mTOR inhibitors, our group and several others have identified mTOR ATP active-site inhibitors, such as PP242 and Torin1 (Feldman *et al*, 2009; Thoreen *et al*, 2009). These compounds reversibly compete with ATP for binding to the mTOR catalytic domain and thus block not only mTORC1 activity, but also mTORC2 activity. mTORC2, an mTOR complex distinct from mTORC1, is responsible for an activating phosphorylation of AKT at Serine 473. Using PP242, our group was the first to demonstrate that these ATP active-site inhibitors effectively inhibit phosphorylation of the 4EBPs, the S6Ks, and AKT. This is in striking contrast to rapamycin, which predominantly blocks the phosphorylation of S6Ks and infrequently blocks the phosphorylation of 4EBPs. As a result, PP242 inhibits the proliferation of cultured cell lines to a much greater extent than rapamycin. Furthermore, our group has shown that PP242 dramatically inhibits tumour growth in an AKT-driven mouse model of lymphoma that is inherently resistant to rapamycin. Strikingly, tumours from the same model that overexpressed a mutated non-phosphorylatable 4EBP1 transgene were completely insensitive to PP242 inhibition, suggesting that PP242 efficacy may be entirely due to its ability to block mTORC1-dependent 4EBP phosphorylation (Hsieh *et al*, 2010). In line with this, PP242 and Torin1 both retain their antiproliferative effects in mouse embryonic fibroblasts, in which the mTORC2 complex has been destabilised (Feldman *et al*, 2009; Thoreen *et al*, 2009). Although these studies suggest that the antitumour effect of mTOR ATP active-site inhibitors is predominantly mediated by blocking phosphorylation of 4EBPs and eIF4E hyperactivity, they cannot generally rule out a role for the inhibition of other translational regulators downstream of mTORC1. Furthermore, it remains to be seen how these ATP active-site inhibitors, which have been found to block growth of cell lines and murine lymphomas, will perform in solid human epithelial tumours.

## Future directions

Oncogenic AKT signalling utilises the multistep process of mRNA translation to drive tumour development and progression. Despite significant advances in our understanding of AKT-mediated translational control and the development of promising therapeutics to target deregulated translation initiation in human cancers, many questions and opportunities remain. Although AKT signalling has been shown to control the translational steps of ribosome biogenesis, translation initiation, and translation elongation, it is still an open question if oncogenic AKT requires the hyperactivation of all three translational steps for tumourigenesis and cancer progression. This is an important question because most preclinical and clinical studies to date have focused on targeting translation initiation downstream of oncogenic AKT. Recently, it has been shown that targeting ribosome biogenesis through pol I inhibition (CX-5461) or targeting various aspects of translation elongation leads to significant antitumour activity *in vivo* (Robert *et al*, 2009; Drygin *et al*, 2011). Thus, it will be important to determine whether more comprehensive inhibition of oncogenic AKT-driven translation through combined targeting of ribosome biogenesis, translation initiation, and translation elongation results in clinically significant improvements in patient survival. Attempts to target translational control downstream of oncogenic AKT will be further aided by the use of novel technologies and analyses to identify networks of translationally controlled genes that may function as critical biomarkers for disease progression and therapeutic response.

Finally, there is a growing body of evidence that deregulation of translational control may be a common mechanism by which oncogenic pathways promote tumour initiation and progression (e.g., MYC and RAS). As such, efforts to target translational control may prove successful in a wide array of human malignancies.

## Figures and Tables

**Table 1:** Common mutations in the PI3K–AKT–mTOR signalling pathway

**Targets**	**Genetic alteration**	**Cancer type**
PIK3CA (phosphoinositide-3-kinase, catalytic, *α*-polypeptide)	Mutations	Breast, endometrial, colon, upper digestive tract, gastric, pancreas, ovarian, liver, brain, oesophageal, lung, melanoma, urinary tract, prostate, thyroid
	Amplifications	Lung (squamous cell), lung (adenocarcinoma), lung (small cell), lung (non-small cell), cervical, breast, head and neck, gastric, thyroid, oesophageal, endometrial, ovarian, glioblastoma
		
PIK3CB (phosphoinositide-3-kinase, catalytic, *β*-polypeptide)	Amplifications	Ovarian, breast
	Increase in activity and expression	Colon, bladder
		
PDPK1 (3-phosphoinositide dependent protein kinase-1)	Amplifications and overexpression	Breast
		
AKT (v-akt murine thymoma viral oncogene homologue)	AKT homologue 1 mutation (E17K) or amplifications	Breast, colon, ovarian, lung, gastric
	AKT homologue 2 amplifications	Ovarian, pancreas, head and neck, breast
	AKT homologue 3 mutation (E17K) or amplifications	Skin, glioblastoma
		
PIK3R1 (phosphoinositide-3-kinase, regulatory subunit-1)	Mutations	Glioblastoma, ovarian, colon
		
PTEN (phosphatase and tensin homologue)	Loss of heterozygosity	Gastric, breast, melanoma, prostate, glioblastoma
	Mutations	Endometrial, brain, skin, prostate, colon, ovary, breast, haematopoietic and lymphoid tissue, stomach, liver, kidney, vulva, urinary tract, thyroid, lung
		
